# Drug-Induced Linear IgA Bullous Dermatosis in an Oncologic Patient

**DOI:** 10.7759/cureus.49185

**Published:** 2023-11-21

**Authors:** Luz A Quispe-Gárate, Renzo B Espinoza-Escudero, Carlos Salas-Rivera, Gadwyn Sánchez-Félix

**Affiliations:** 1 Medicine, Universidad Nacional Mayor de San Marcos, Lima, PER; 2 Dermatology, Hospital Nacional Edgardo Rebagliati Martins, Lima, PER

**Keywords:** bullous dermatosis, clinical dermatology, cutaneous adverse drug reaction, vancomycin reaction, linear iga bullous dermatosis

## Abstract

Blister formation in the skin can result from various conditions, such as autoimmune disorders, drug reactions, infections, etc. A comprehensive patient assessment may offer clues for diagnosis. Linear IgA bullous dermatosis (LABD) is a rare subepidermal blistering disorder characterized by the deposition of IgA at the basement membrane zone of the skin and mucous membranes. Here, we describe a case of a patient with a new onset of painless blisters located in the skin and oral mucosa after initiating antibiotic treatment.

## Introduction

Linear IgA bullous dermatosis (LABD) is a rare skin disease due to IgA autoantibodies against the basement membrane zone. It has an incidence rate of 0.2 to 0.4 per million per year, with two peaks in childhood and adulthood, respectively. Diagnosis is made based on clinical, histopathological, and immunological features. It classically presents with clear or hemorrhagic vesicles and blisters with a widespread distribution in the extremities, trunk, buttocks, and face, some with an annular pattern. Mucosal involvement can also occur with blisters, erosions, and ulcerations, mainly in the oral cavity and genital areas [1].

Although LABD is commonly described as an idiopathic disease, some precipitating factors have been identified such as drugs, systemic diseases (such as non-Hodgkin lymphoma, chronic lymphocytic leukemia, ulcerative colitis, and systemic lupus erythematosus), and trauma [1]. Here, we report a case of drug-induced LABD after administering vancomycin therapy.

## Case presentation

**Figure 1:**
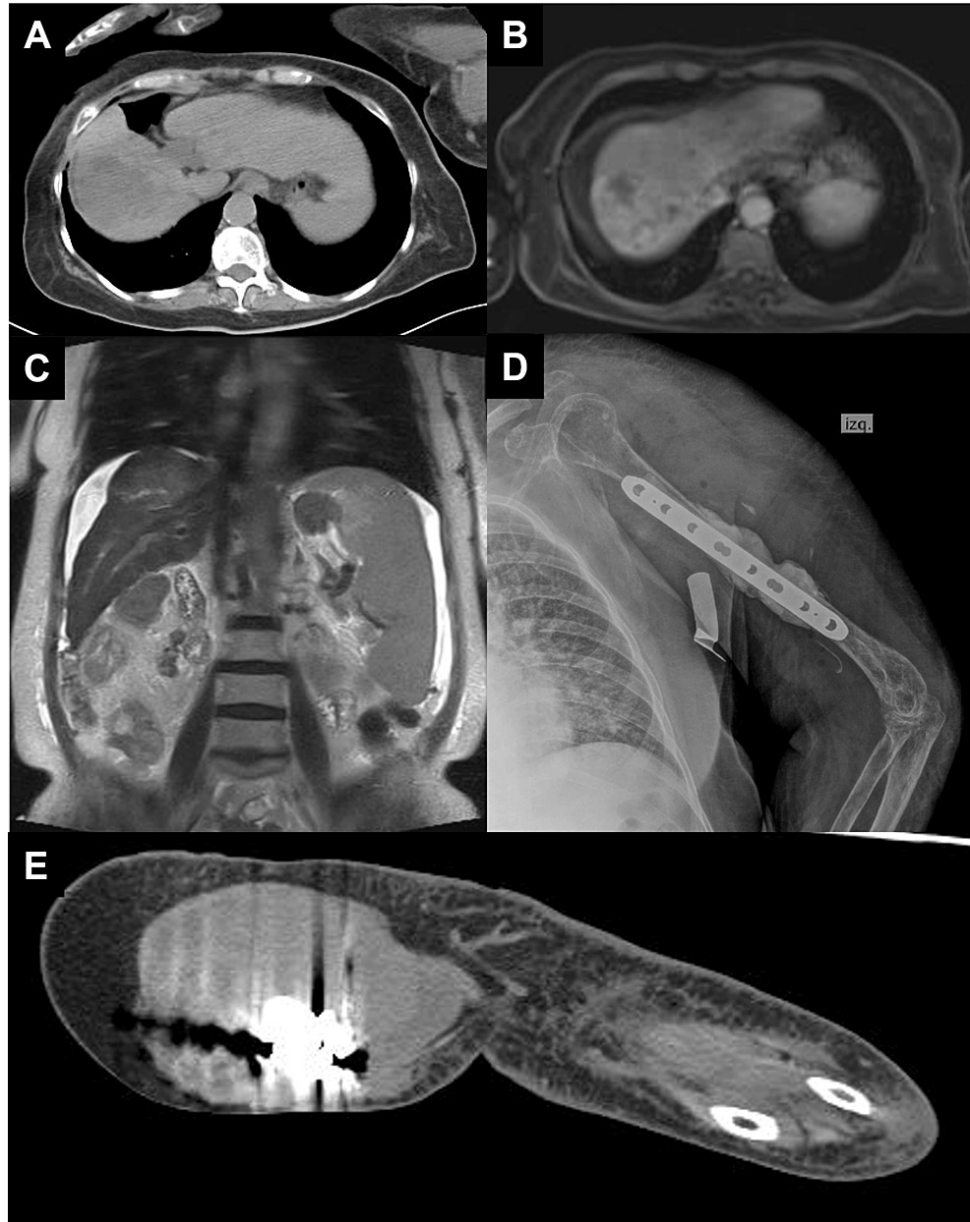
Radiologic findings (A-C) An 85x65 mm heterogeneous, solid mass with a wedge-shaped morphology located in the hepatic segments VII and VIII. It partially surrounds the right suprahepatic vein near the inferior vena cava, without infiltration of its lumen. Splenomegaly is also appreciated. (D) Left upper arm X-ray showing osteosynthesis of humerus shaft with fracture callus. (E) In the axial plane, there is a neoformative process in the distal-middle third of the left arm that measures 10x10 cm. (A, E: Multislice spiral CT without contrast) (B, C: Contrast-enhanced MRI).

A 69-year-old woman presented to the Emergency Department with a six-month history of edema, erythema, and pain in her left arm. Her previous medical history includes cirrhosis, retinal detachment, and a recent diagnosis of hepatic carcinoma with metastatic involvement of the bone and lungs. She was not on chemotherapy at presentation. Tumoral process was identified following a pathologic fracture of the left humerus which was treated with osteosynthesis (Figure 1). She reported being allergic to penicillin and iodine.

Physical examination revealed a temperature of 36 degrees Celsius, blood pressure of 120/60 mmHg, heart rate of 91 beats per minute, respiratory rate of 17 breaths per minute, and oxygen saturation of 98% on room air. Laboratory analysis showed hemoglobin of 11.26 g/dL (reference range: 12-16 mg/dL), white blood cell count of 2.06 x 10^3^/uL (4-10 x 10^3^/uL), neutrophils 1.6 x 10^3^/uL (1.5-7.5 x 10^3^/uL), lymphocytes 0.3 x 10^3^/uL (1-5.2 x 10^3^/uL), platelets 42.4 x 10^3^/uL (150-450 x 10^3^/uL), urea of 17 mg/dL (10-50 mg/dL), creatinine of 0.53 mg/dL (0.5-1.4 mg/dL), C-reactive protein of 1.2 mg/dL (<0.5 mg/dL), and procalcitonin of 0.194 ng/mL (<0.065 ng/mL).

**Figure 2:**
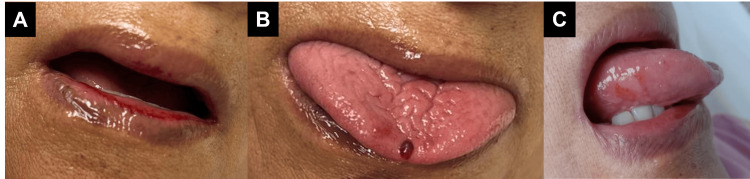
Lesions in oral mucosa (A) Erosions of the vermillion border of both lips; (B) Tense hemorrhagic blister on the tip of the tongue; (C) Erythematous erosions on a white background in the lateral aspect of the tongue.

**Figure 3:**
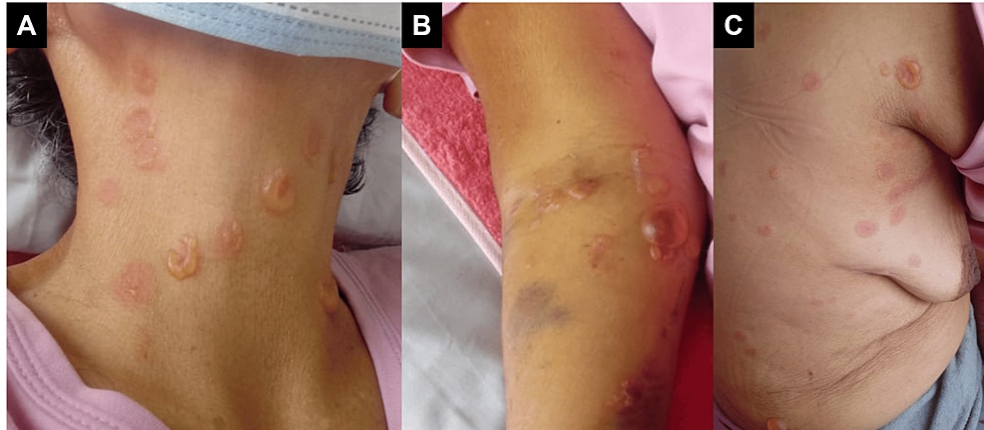
Lesions in skin Multiple tense bullae across the neck (A), arm (B), and back (C), some of them showing a string-of-pearls appearance.

She was admitted under the diagnosis of possible cellulitis, and antibiotic coverage with vancomycin was started (1 g IV every 12 hours). She presented a generalized bullous skin eruption on the ninth day of treatment. Physical examination revealed erosions on the oral mucosa and multiple tense vesiculobullous lesions across the neck, chest, abdomen, back, arms, and legs in an arciform or annular configuration with a string-of-pearls appearance (Figure 2, 3). Nikolsky and Asboe-Hansen signs were negative. Antibiotic use was suspended, and lesional and perilesional punch biopsies were taken from a blister on the chest for H&E staining and direct immunofluorescence (DIF) testing.

**Figure 4:**
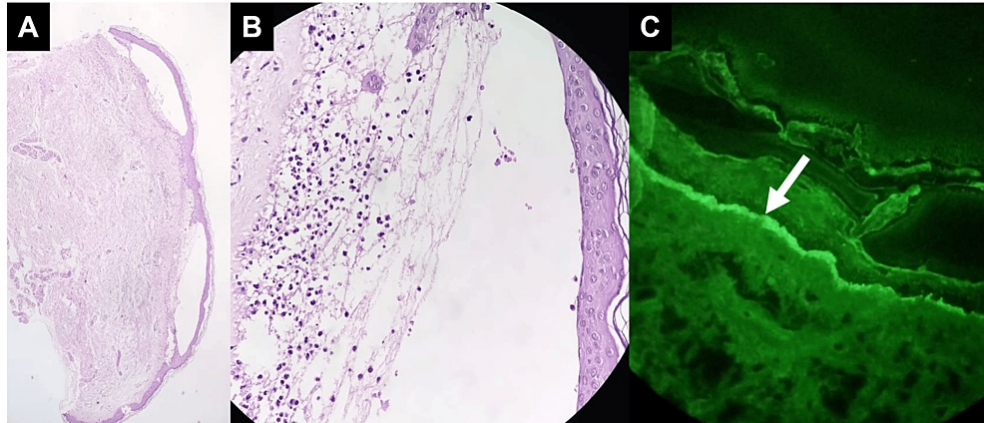
Histopathological and direct immunofluorescence findings (A) Histology specimen showing subepidermal blisters (H&E, x40); (B) There is an inflammatory infiltrate in the upper dermis composed mainly by neutrophils (H&E, x200); (C) Biopsy specimen from perilesional skin revealing linear IgA deposits in the basement membrane zone (arrow) (direct immunofluorescence, x200).

**Figure 5:**
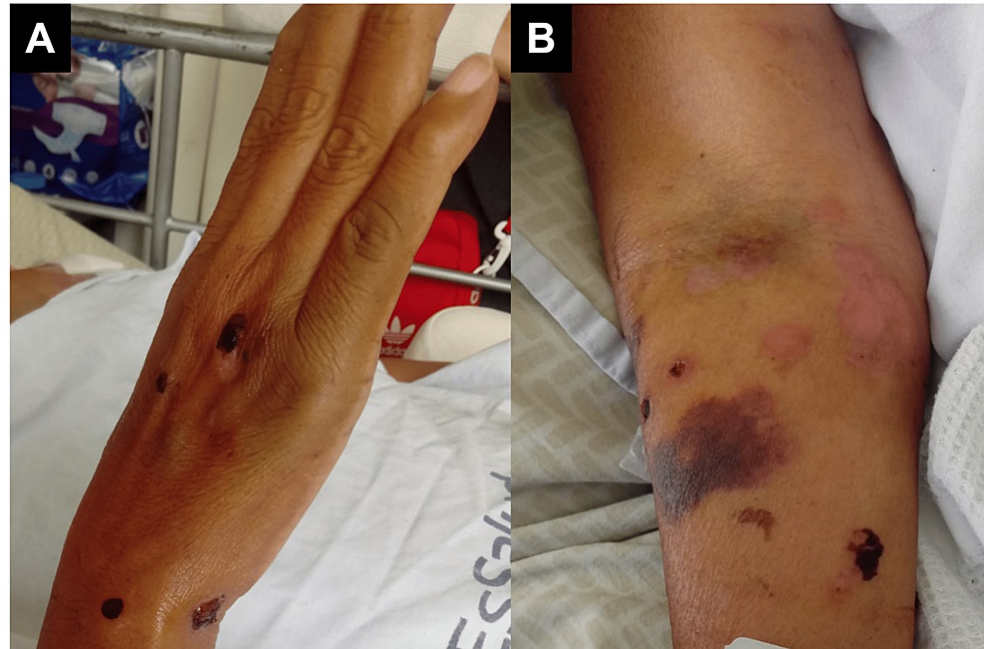
(A, B) Improvement of skin lesions one week after the withdrawal of vancomycin

Based on the patient’s history, vancomycin-induced LABD, paraneoplastic pemphigus, and bullous pemphigoid were considered differential diagnoses. Histopathologic findings revealed a subepidermal blister with a neutrophilic infiltrate in the upper dermis. There was no evidence of apoptotic keratinocytes or interface dermatitis. In DIF, a linear band of IgA at the basement membrane zone was found (Figure 4). A diagnosis of vancomycin-induced LABD was made. Five days after withdrawing the antibiotic, there was no appearance of new lesions. The complete recovery was achieved two weeks later without further treatment (Figure 5).

## Discussion

LABD is a skin disorder due to the deposition of IgA autoantibodies in the basement membrane zone. It is commonly described as an idiopathic blistering disorder, but a drug-induced variant has been reported. Pediatric and adult populations are affected, with a mean age of diagnosis of 5.4 and 60.6 years, respectively. It usually manifests with vesiculobullous lesions on an erythematous base involving the limbs, trunk, head, or buttocks. Mucosal involvement has also been described, with oral, genital, and conjunctival lesions in order of frequency [2]. Other common findings include a string-of-pearls pattern, erythematous plaques, erosions, and target-like lesions [2,3].

Pathogenesis is not clearly understood. IgA autoantibodies bind to different hemidesmosomal antigens. Antibody targets include bullous pemphigoid 180-kDa (BP180), 97-kDa protein (LABD-97), 120-kDa protein (LAD-1) (both extracellular domains of BP180), type VII collagen, laminin 332 and bullous pemphigoid 230 kDa antigen (BP230). These target proteins can be divided into two categories: lamina lucida and sub-lamina densa types. LAD-1 and LABD-97 are the most common targets in the lamina lucida type. In contrast, type VII collagen has been identified as the main target in sub-lamina densa type [4,5].

Drug-induced LABD was first described in 1981 in a woman under diclofenac therapy [6]. Since then, many other drugs have been linked to this variant. Vancomycin has been identified as the most common triggering factor (46.2%) [7]. However, it can be associated with amoxicillin, amoxicillin-clavulanic acid, imipenem, metronidazole, ketoprofen, enoxaparin, verapamil, atorvastatin, etc. The median time from first drug intake to disease onset is nine days. Compared to idiopathic LABD, erosions and Nikolsky sign are more common in drug-induced LABD, with a higher frequency of lesions mimicking toxic epidermal necrolysis (TEN) when vancomycin is identified as the root cause [8]. Although our patient had a negative Nikolsky sign, the onset and disease localization were consistent with the literature.

In H&E staining, there is evidence of subepidermal blisters with neutrophilic infiltration in the papillary dermis. However, this feature is not pathognomonic as it is also visualized in bullous lupus erythematosus, dermatitis herpetiformis, and bullous vasculitis [9]. Additional findings may include eosinophilic infiltration of the upper dermis, neutrophil microabscesses in the dermal papillae, and necrotic keratinocytes. The most important diagnostic feature in DIF is a linear IgA deposition in the basement membrane zone, either alone or accompanied by IgG, IgM, or complement deposits [2,10].

Management is centered on drug discontinuation. Dapsone is considered the first-line therapy for both adult and pediatric populations. Treatment can be started at low doses (25 mg/day) and titrated up over time based on clinical condition (50-150 mg/day). In children, doses range from 0.5-2 mg/kg/day. Dapsone-induced hemolysis is a serious adverse effect reported in people with glucose-6-phosphate deficiency (G6PD). Therefore, patients must be screened for G6PD before starting drug administration. Sulfonamides (sulfapyridine or sulfamethoxypyridazine) are considered alternative agents that can be used alone or combined with dapsone. Suldapyridine dose ranges from 1000-1500 mg/day, in adults, and 15-60 mg/kg/day, in children [11]. Other therapeutic options include rituximab, IV immunoglobulin, topical and systemic corticosteroids, colchicine, cyclosporine, nicotinamide, and tetracyclin. Depending on the skin and mucosal involvement, these agents can be used alone or in combination, and frequency and dosing vary depending on clinical criteria. In recent years, omalizumab and etanercept have also been used as alternative agents [12].

LABD has a good prognosis. The remission rate ranges from 75-81% in the idiopathic and drug-induced variants [2,8]. The mean time to complete clinical remission is 13 days (1-21 days) in vancomycin-induced LABD, and 2.7 weeks (1-5 weeks) in drugs other than vancomycin [7].

Finally, vancomycin has been linked to a variety of immune-mediated reactions. Type IV hypersensitivity reactions include, in order of frequency, LABD, drug reaction with eosinophilia and systemic symptoms (DRESS) syndrome, Stevens-Johnson syndrome, and TEN [13]. We emphasize the importance of identifying drug-induced cutaneous reactions, especially in patients with multiple comorbidities and a new onset of blistering lesions.

## Conclusions

This case report emphasizes the importance of integrating clinical and pathological features when diagnosing bullous dermatosis. LABD is a rare entity that can be triggered by drug intake that leads to blister formation in skin and oral, genital, and conjunctival mucosa. Lesions usually improve after discontinuing drug treatment, but some agents like vancomycin may increase the risk of developing a life-threatening skin reaction. Therefore, proper monitoring should be provided for early diagnosis and adequate treatment.
